# [Retracted] SDF1/CXCR4 axis facilitates the angiogenesis via activating the PI3K/AKT pathway in degenerated discs

**DOI:** 10.3892/mmr.2024.13331

**Published:** 2024-09-16

**Authors:** Hanxiang Zhang, Peng Wang, Xiang Zhang, Wenrui Zhao, Honglei Ren, Zhenming Hu

Mol Med Rep 22: 4163–4172, 2020; DOI: 10.3892/mmr.2020.11498

Following the publication of this paper, it was drawn to the Editors’ attention by a concerned reader that certain of the Transwell invasion assay data shown in Figs. 2E, 3E, 4E and 5E, and the Transwell migration assay data shown in Fig. 2D, were strikingly similar to data appearing in different form in other articles written by different authors at different research institutes that had either already been published elsewhere prior to the submission of this paper to *Molecular Medicine Reports*, or were under consideration for publication at around the same time (some of which have already been retracted). Moreover, data were also found to be duplicated comparing the data panels in Figs. 3D and 4D, such that data which were intended to have shown the results from differently performed experiments had been derived from the same original source. In view of the fact that certain of the abovementioned data had already apparently been published previously, the Editor of *Molecular Medicine Reports* has decided that this paper should be retracted from the Journal. The authors were asked for an explanation to account for these concerns, but the Editorial Office did not receive a reply. The Editor apologizes to the readership for any inconvenience caused.

